# Discrimination of persons living with obesity by healthcare professionals

**DOI:** 10.23938/ASSN.1161

**Published:** 2026-04-20

**Authors:** José I. Baile, María José González-Calderón, María Frenzi Rabito-Alcón

**Affiliations:** Facultad de Ciencias de la Salud Universidad a Distancia de Madrid (UDIMA) Madrid España

**Keywords:** Discrimination, Obesity, Healthcare, Obesity Bias, Healthcare Professionals, Discriminación, Obesidad, Atención Sanitaria, Sesgo por obesidad, Profesionales Sanitarios

## Abstract

Obesity is a highly prevalent and complex condition with biomedical, psychological, and social dimensions that pose major challenges for healthcare systems. Beyond its clinical consequences, people living with obesity frequently experience weight-based discrimination in healthcare settings, undermining care quality and equity. This paper examines key mechanisms underlying obesity-related stigma among healthcare professionals, including attribution of excess weight to individual responsibility and the internalization of stigma by patients. Drawing on current evidence, it analyses how negative attitudes of healthcare professionals contribute to suboptimal clinical interactions, inadequate care environments, and reduced patient engagement. These processes may weaken the therapeutic relationship, hinder treatment adherence, and negatively affect physical and mental health outcomes. Addressing obesity stigma requires targeted professional training, adaptation of care environments, and inclusive health policies to ensure equitable and respectful care for this population.

## RESUMEN

La obesidad es una condición de salud altamente prevalente y compleja, con implicaciones biomédicas, psicológicas y sociales. Las personas con obesidad experimentan con frecuencia en entornos asistenciales discriminación basada en el peso, comprometiendo la calidad y la equidad de la atención. Examinamos los principales mecanismos que sustentan el estigma asociado a la obesidad entre profesionales sanitarios, incluidos la atribución del exceso de peso a la responsabilidad individual y la internalización de estos prejuicios por los propios pacientes. Se analiza cómo las actitudes negativas pueden traducirse en interacciones clínicas subóptimas, entornos asistenciales inadecuados y menor implicación del paciente. Estas dinámicas debilitan la relación terapéutica, dificultan la adherencia al tratamiento y afectan negativamente los resultados de salud física y mental. Abordar el estigma de la obesidad requiere capacitación profesional específica, adaptación de los entornos de atención, y políticas de salud inclusivas para garantizar una atención equitativa y respetuosa para esta población.

Obesity is a health condition characterised by an excess of fat mass relative to total body weight, which can lead to significant biomedical, psychological, and social repercussions. According to the World Health Organisation, the global prevalence of obesity has reached such a scale that it is now considered an epidemic^1^. In Spain, the prevalence of excess weight approaches half of the adult population, with obesity itself affecting one in five individuals, according to the Nutritional Study of the Spanish Population^2^. These figures suggest that obesity represents a major challenge for healthcare systems, requiring considerable medical attention, and that any medical or psychosocial issues related to it affect a substantial portion of the population. For instance, obesity is strongly comorbid with various psychopathological disorders, such as those linked to depression or anxiety^3^. Consequently, the study, prevention, and treatment of these issues should be a priority.

One of the psychosocial consequences of obesity that requires particular attention is the discrimination experienced by people living with obesity, which is the focus of this paper. Specifically, these persons are often attributed more negative personal traits than their non-obese counterparts, such as being lazy, sluggish, or less intelligent^4^. These prejudices, alongside the stigmatisation of their condition, provide the basis for discrimination, which limits their rights and opportunities in various areas, such as education, the workplace, and healthcare. In the healthcare domain, however, individuals with obesity may encounter an even more troubling form of discrimination, as it is precisely the healthcare professionals they rely on to address their health issues who are expected to demonstrate understanding of their circumstances and offer full acceptance as patients, rather than perpetuating discrimination based on their obesity.

A fundamental concept underlying obesity-related stigma among healthcare professionals is the insufficient recognition of obesity as a chronic, multifactorial disease. Obesity should not be understood merely from a therapeutic standpoint, but as an essential etiopathogenic condition shaped by complex interactions among genetic, neurobiological, metabolic, environmental, and psychosocial factors. Failure to acknowledge this chronic disease framework may reinforce simplistic attributions of personal responsibility and contribute to weight bias in clinical practice.

For the reasons outlined above, the objectives of this study are (a) to review the current scientific evidence on discrimination towards individuals with obesity by healthcare professionals and, (b) to analyse the potential causes and consequences of such discrimination, with the aim of proposing measures for its reduction, alongside strategies for its prevention and elimination.

Although this study does not follow a systematic review methodology, a structured, non-systematic literature search was conducted to identify relevant publications on obesity-related stigma in healthcare settings. The search was performed in major databases, including Web of Science and PubMed, using keywords such as “*obesity stigma*”, “*weight bias*”, “*healthcare professionals*”, and “*discrimination*”. In addition, a snowball strategy was applied by examining the references of selected articles. After screening for relevance and excluding non-pertinent sources (e.g., non-scientific texts and books), a total of 42 references were included in this review.

## OBESITY DISCRIMINATION: MAIN CAUSES AND CONTEXTS IN WHICH IT OCCURS

In many Western societies, women are expected to maintain a slender appearance in line with prevailing beauty standards, while men are encouraged to display an athletic or muscular physique. In both cases, individuals with excess weight are positioned outside what is socially considered an acceptable or attractive body, which favours stigmatisation. As a result, people living with obesity are frequently ascribed negative personal attributes without factual basis, giving rise to a halo effect that leads to their social devaluation. For instance, children with obesity are often labelled as lazy, careless, dirty, dishonest, aggressive, unattractive, or unintelligent^5^. These stereotypes constitute a major source of stigma and discrimination, although several additional factors contribute to their persistence.

One key factor is the widespread belief in the controllability of obesity, often linked to attributions of personal responsibility and lack of self-control^6^. Another contributing mechanism is the internalisation of these prejudices by individuals with obesity themselves, who may progressively accept such beliefs, adopt behaviours consistent with negative stereotypes^7^, and resign themselves to discriminatory treatment rather than actively challenging it. A further argument sustaining stigma is the notion that discrimination occurs for *the person’s own good*^8^, based on the assumption that shame or guilt may motivate weight loss. However, evidence indicates that stigmatisation does not promote healthy behavioural change and may instead increase health risks and hinder weight management efforts^9^. Additionally, the underrepresentation of individuals with obesity in leadership positions and in media or cultural representations reinforces negative stereotypes while privileging alternative aesthetic ideals, thereby perpetuating stigma and discrimination^10^.

Since the late 20th century, numerous studies have documented the stigmatisation of obesity and its discriminatory consequences across multiple social contexts. Discrimination has been reported in the workplace, affecting recruitment processes^11^, professional relationships^12^, and employment stability^13^. Similarly, stigma towards overweight children and adolescents has been observed in educational settings, both from teachers and peers^14^. Negative attitudes have also been consistently identified in healthcare contexts among professionals such as physicians, nurses, dietitians, psychologists, and medical students^15^.

In addition, obesity-related stigma does not operate in isolation. An intersectional perspective highlights that weight-based discrimination often overlaps with other forms of social disadvantage, such as gender, race, ethnicity, or socioeconomic status. These intersecting identities may amplify the negative consequences of stigma, leading to compounded experiences of marginalisation and unequal access to resources, including healthcare. Recognising this intersectionality is essential to fully understand the structural dimensions of weight stigma and to design more equitable and inclusive interventions.

## OBESITY DISCRIMINATION IN HEALTHCARE SETTINGS

The existence of stigmatisation and discrimination towards individuals with obesity in healthcare settings, whether explicit or implicit, has been extensively documented since the pioneering study by Maddox and Liederman^16^. In 1969, they found that doctors and medical students perceived patients with obesity as being stupid (97%), failures (90%), weak (90%), lazy (86%), unpleasant (69%), unhappy (65%), lacking willpower (60%), clumsy (55%), and unattractive (54%). This stigmatisation has been observed not only among doctors but also among nurses, nutritionists, mental health professionals, and, more concerningly, even specialists in the treatment of obesity^17^. Among healthcare professionals, the prejudice regarding the controllability of obesity is particularly widespread^18^. Many healthcare professionals attribute obesity primarily to individual factors and interpret treatment outcomes as largely dependent on patient adherence. Furthermore, they often emphasise the emotional origins of this health issue. In primary care settings, condescending attitudes and disrespect towards individuals with obesity have also been reported, with a tendency to attribute all their health problems to excess weight, alongside a notable lack of adequate training among these professionals^19^. Moreover, among psychology professionals, a negative halo effect appears to be prevalent, where they assume that individuals with obesity experience greater psychopathological symptoms than their non-obese counterparts^20^.

Even the physical infrastructure of healthcare settings can contribute to the exclusion and stigma experienced by people with obesity, limiting their access to essential services and adequate medical care due to the significant barriers posed by a lack of adapted resources. Among the issues identified are the absence of wide and appropriately spaced waiting chairs, accessible and durable examination tables, correctly sized blood pressure cuffs, and equipment not designed for individuals with obesity, such as magnetic resonance imaging (MRI) machines. In the field of gynaecological care, a study revealed that 46% of women with obesity reported small gowns, narrow examination tables, and inadequate equipment as obstacles to receiving quality healthcare^21^. Additionally, 35% of these women reported feeling shame when being weighed.

## CONSEQUENCES OF OBESITY DISCRIMINATION IN HEALTHCARE SETTINGS

Obesity discrimination in healthcare settings leads to consequences across various domains. Firstly, patients with obesity are often able to recognise these negative attitudes and behaviours from healthcare professionals. They feel that they are treated with less respect and receive poorer care compared to non-obese individuals, which generates distrust and lower satisfaction with healthcare services and professionals^22^, as well as a sense of being in an unsafe or unfriendly environment^23^. For example, in a study with bariatric surgery patients, participants reported feeling mistreated by the healthcare professionals involved in their treatment^24^. This perception has been confirmed in several studies, which found that healthcare professionals tend to provide inferior care to patients with obesity, whether in terms of the time spent on consultations, the development of a comprehensive medical history, or the quality of treatment plans^25^.

This stigmatisation within healthcare settings has also been linked to poorer health outcomes among patients with obesity, as it negatively influences health behaviours such as diet and physical activity and may even contribute to the worsening of certain clinical indicators, including blood pressure^26^. It has been observed that these individuals are more likely to delay seeking preventive healthcare services^19^, such as mammograms^27^, tend to cancel medical appointments more frequently^28^, and are less motivated to follow therapeutic guidelines^25^. Moreover, the stigma surrounding obesity in healthcare has detrimental effects on patients’ mental health, with common reports of feelings of shame and lowered self-esteem. This can create a vicious cycle in which these patients delay seeking care, leading to more severe complications and thereby increasing treatment costs and complexity. For these reasons, weight-based discrimination is widely recognised as a critical barrier to the provision of high-quality care for individuals with obesity.

## CAUSES OF OBESITY DISCRIMINATION IN HEALTHCARE SETTINGS

**Figure 1:**
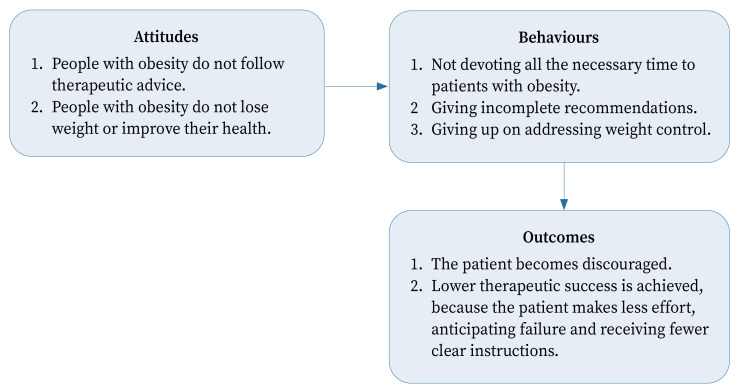
Interaction between negative attitudes and behaviours of healthcare professionals towards individuals with obesity (adapted from Fabricatore et al., 2005).

Although there is no singular, comprehensive theory explaining obesity discrimination in healthcare settings, beyond the societal factors already discussed, several interaction models have been developed to shed light on how the stigmatisation and discrimination of obesity among healthcare professionals are both justified and perpetuated. One such model is proposed by Fabricatore et al.^29^. According to this model of interaction between attitudes and behaviours, negative attitudes held by healthcare professionals towards individuals with obesity influence their behaviour, leading to suboptimal care and poor health outcomes for these individuals. These negative outcomes, in turn, reinforce the initial prejudiced attitudes, creating a vicious cycle, as illustrated in Figure 1.

According to this model, healthcare professionals may exhibit a form of neglect or *therapeutic nihilism* towards patients with obesity, which ultimately leads to poorer therapeutic outcomes^29^. In turn, these suboptimal outcomes result in patients becoming less engaged in their treatments, reinforcing the initial perception among healthcare professionals that these patients are uncommitted. This, in turn, leads to a reduced effort on the part of professionals to address their needs. While this model requires further in-depth study to confirm the variables it encompasses and the relationships between them, some of its hypotheses have been corroborated in studies such as that by Jay et al.^30^, where 40% of doctors treating patients with obesity reported negative reactions and frustration in their care.

Although figure 1 presents the core interaction model originally proposed by Fabricatore et al.^29^, the mechanisms involved in obesity-related stigma in healthcare settings may be conceptually expanded. In particular, weight bias among healthcare professionals can contribute to stigmatizing clinical interactions that foster internalization of stigma in patients. This internalization may activate chronic psychological stress pathways, increasing vulnerability to psychiatric symptoms, maladaptive coping strategies such as binge eating, reduced adherence to therapeutic recommendations, and worsening obesity-related complications. These downstream effects may, in turn, reinforce professionals’ negative expectations, perpetuating the cycle.

## CONCLUSIONS

The reviewed literature suggests that stigma and discrimination towards patients with obesity in healthcare settings, driven by persistent biases, contribute to suboptimal medical care and exacerbate disparities in both access to care and the quality of treatment^15^^-^^17^. These biases make it more difficult for patients to seek help and follow medical advice^31^^,^^32^, aggravate comorbidities commonly associated with obesity, and ultimately negatively affect the quality of the care received^21^^,^^23^. These negative perceptions are largely rooted in reductionist views that attribute obesity primarily to individual responsibility, thereby overlooking its multifactorial and chronic nature^33^. Such clinical bias not only perpetuates a hostile environment but also compromises the quality of care, underscoring the need for a more comprehensive approach^34^. In practical terms, this approach should include the integration of stigma-reduction modules in healthcare training programmes, the development of clinical guidelines promoting respectful obesity care, and the systematic use of person-first language in clinical settings. Despite this, the implications of stigma and discrimination by healthcare professionals have often been underestimated, highlighting the need for a more inclusive approach to healthcare services^35^.

Indeed, it is now recognised that a broader and more inclusive etiological and clinical approach is necessary to properly understand obesity and effectively address its prevention and treatment^36^^,^^37^. In fact, obesity should be regarded as a multifactorial condition^33^^,^^38^, not solely as an individual responsibility, and it should be treated as a chronic disease rather than an isolated issue that can be resolved through one-time interventions, such as a restrictive diet. This perspective is reinforced by the recent American Association of Clinical Endocrinology (AACE) consensus statement^39^ on weight stigma, which formally recognises obesity as a chronic, relapsing disease and explicitly calls for the elimination of weight bias across healthcare systems. The consensus highlights the ethical responsibility of healthcare professionals to adopt person-centred, evidence-based, and non-stigmatising approaches in obesity care.

Furthermore, some patients with obesity may have a history of therapeutic failures and a lack of understanding of their condition, which healthcare professionals should consider in order to better comprehend their health problems and provide appropriate care. At this point, it is important to highlight the growing ethical debate surrounding whether patients who require medical care for conditions derived from personal decisions, such as poor lifestyle habits, should receive the same level of care as those whose conditions are not attributed to individual responsibility. This distinction is overly simplistic, as many diseases that may appear unrelated to individual behaviour could, in fact, be influenced by lifestyle habits. It is crucial to reconsider judgments about individual responsibility in the genesis of diseases, given the multiple environmental and biological factors that can influence health^35^^,^^40^^,^^41^.

## FUTURE ACTIONS

### 1. Education and awareness about obesity in healthcare settings

Weight bias among healthcare professionals is one of the most significant drivers of stigmatizing attitudes toward people living with obesity. This bias is frequently rooted in insufficient training and in a limited understanding of obesity as a chronic, multifactorial disease. When physicians and nurses lack adequate education regarding the complex etiopathogenesis and long-term management of obesity, they may be more likely to attribute excess weight primarily to personal responsibility. Therefore, obesity education should be urgently strengthened across all stages of professional development, from undergraduate medical and nursing curricula to postgraduate training and continuing professional education, including primary care and other medical specialties.

In this context, it is crucial to implement continuous training programmes that explicitly address obesity-related biases and discrimination, as supported by various scholars, such as Talumaa^36^. These programmes should aim to move beyond the simplistic view of obesity as merely a condition under the patient’s control, emphasising its multifactorial causes and the need for an integrated, chronic care approach. Additionally, they should incorporate communication skills training that promotes a more empathetic doctor-patient relationship^40^, which may significantly improve treatment adherence and the overall quality of care.

In addition to theoretical training, structured clinical frameworks can facilitate non-stigmatising obesity care in everyday practice. The Modified 5 As approach (Ask, Assess, Advise, Agree, Assist) has been proposed^42^ as a practical strategy for obesity counselling in primary care. This framework promotes respectful communication, shared decision-making, and collaborative goal setting, thereby reducing the likelihood of weight-biased interactions.

From a practical perspective, training programmes should include specific components such as raising awareness of implicit bias, promoting the use of person-first language, and implementing structured communication strategies for discussing weight sensitively. Techniques such as role-playing, reflective practice, and feedback-based learning may enhance healthcare professionals’ capacity to manage clinical interactions in a non-stigmatising manner.

### 2. Adaptation of healthcare infrastructure

Adapting healthcare infrastructure and medical equipment in healthcare centres is a critical step towards removing physical barriers that hinder the care of patients with obesity. The introduction of appropriately sized chairs, examination tables, and diagnostic equipment, such as MRI machines and correctly sized blood pressure cuffs, would help reduce exclusion and significantly enhance the patient experience. These improvements would make healthcare centres more inclusive, preventing situations of shame or discomfort that could affect patients’ willingness to seek care. In addition, regular assessments of healthcare environments and the incorporation of patient feedback mechanisms may help identify structural barriers and guide improvements in accessibility and inclusivity.

### 3. Research on the causes and effects of obesity stigma

Further research is required to gain a deeper understanding of how stigma associated with obesity impacts both the healthcare patients receive and the outcomes of interventions on patients’ health. Specifically, studies should investigate how perceived negative treatment affects patients’ mental health and their willingness to seek preventive care. Additionally, research evaluating specific interventions to reduce stigma in clinical settings could provide valuable insights into improving the relationship between healthcare professionals and patients with obesity.

### 4. Promotion of inclusive health policies

Public health policies aimed at enhancing the relationship between patients and healthcare professionals should promote the following:

- Awareness campaigns that explicitly address weight stigma and promote evidence-based understanding of obesity;
- Implementation of institutional protocols ensuring respectful communication and equitable treatment of patients with obesity across all levels of healthcare;
- Development of multidisciplinary programmes integrating medical, psychological, and behavioural approaches, while avoiding punitive or weight-centred strategies.

## FINAL REFLECTION

The available evidence consistently indicates that weight-based stigma in healthcare settings constitutes a significant barrier to the provision of equitable and effective care for individuals living with obesity. Negative attitudes among healthcare professionals may compromise the therapeutic relationship, reduce patient engagement, and worsen both psychological and clinical outcomes. Addressing this issue requires a comprehensive approach that includes enhanced professional education, the adoption of non-stigmatising communication strategies, and structural adaptations within healthcare systems. Promoting a more inclusive and evidence-based perspective on obesity is essential to improving the quality of care and health outcomes in this population.

## Data Availability

Not applicable. No datasets were generated or analyzed in this study.
